# Circulating Fibroblast Growth Factor 23 Is Associated with Angiographic Severity and Extent of Coronary Artery Disease

**DOI:** 10.1371/journal.pone.0072545

**Published:** 2013-08-28

**Authors:** Yunjun Xiao, Chaoqiong Peng, Wei Huang, Jinzhou Zhang, Min Xia, Yuan Zhang, Wenhua Ling

**Affiliations:** 1 Department of Nutrition and Food Hygiene, Shenzhen Centre for Disease Control and Prevention, Shenzhen, Guangdong, China; 2 Guangdong Provincial Key Laboratory of Food, Nutrition and Health, Sun Yat-sen University, Guangzhou, Guangdong, China; 3 Department of Nutrition, School of Public Health, Sun Yat-sen University, Guangzhou, Guangdong, China; 4 Department of Cardiology, Guangzhou Military General Hospital, Guangzhou, Guangdong, China; Brigham and Women’s Hospital, Harvard Medical School, United States of America

## Abstract

**Objective:**

Fibroblast growth factor 23 (FGF23) is a circulating regulator of phosphate and vitamin D metabolism and is associated with coronary artery calcification, and has been implicated in the pathogenesis of cardiovascular disease. The aim of this study was to determine whether circulating FGF23 concentration is independently associated with the severity and extent of coronary artery disease in patients undergoing coronary angiography.

**Method:**

A cross-sectional design was used to examine the relationship between serum FGF23 and the severity and extent of coronary artery stenosis in 2076 patients undergoing coronary angiography (1263 male and 813 female, mean aged 62.5 years). Subgroup analyses were performed to assess the associations between FGF23 and coronary arterial plaque characteristics evaluated by intravascular ultrasound and 12-month incidence of target vessel revascularization (TVR) and target lesion revascularization (TLR).

**Findings:**

We found a stepwise increase of serum FGF23 concentrations in patients with mild, moderate, severe stenosis or with increased number of stenotic vessels compared with those without stenosis (*P*<0.001). Serum FGF23 concentration was positively correlated with stenosis scores as the global index of the severity and extent of coronary artery stenosis in both male and female (*r* = 0.315 and *r* = 0.291, *P*<0.001). In multiple regression analyses, serum FGF23 concentration was a significant determinant of the stenosis scores independent of other traditional risk factors (standardized *β* = 0.326, *P*<0.001). Furthermore, subgroup analyses found FGF23 was significantly associated with plaque and dense calcium volumes. Multiple logistic regression analyses showed that serum FGF23 levels were significantly independent predictors of TVR and TLR.

**Conclusions:**

We report an independent association between circulating FGF23 concentration and the severity and extent of coronary artery stenosis in the coronary angiographic patients. Future studies are needed to elucidate the potential biological mechanisms and whether FGF23 is a modifiable cardiovascular risk factor.

## Introduction

Fibroblast growth factor 23 (FGF23) is a recently discovered 30 kDa bone-derived circulating hormone that plays an important role in the complex and tightly regulated mechanism of mineral metabolism, including hyperphosphatemia, vitamin D insufficiency, and elevated parathyroid hormone (PTH) levels [1]. FGF23 acts through one of the FGF receptors, with klotho as a co-receptor, to inhibit renal phosphate reabsorption and decrease circulating levels of 1,25(OH)_2_D and inhibit PTH secretion by the parathyroid glands [2], [3]. Alterations in mineral metabolism are frequently present in chronic kidney disease (CKD) and have been implicated as risk factors for cardiovascular morbidity and mortality in these patients [4]. Because of the important role of FGF23 in mineral metabolism it may also affect cardiovascular risk.

Increased serum FGF23 concentrations were associated with adverse outcomes such as increased mortality in patients undergoing hemodialysis and mortality and cardiovascular events in patients with coronary artery disease (CAD) [5], [6], [7]. Elevated FGF23 levels have been associated with left ventricular hypertrophy in CKD patients [8]. Recently, higher FGF23 levels have been found to be associated with vascular dysfunction [9], total body atherosclerosis [10], and prevalent cardiovascular disease in elderly individuals with normal renal function in community [11]. However, other two studies reported that circulating FGF23 did not correlate with the development of incident coronary heart disease or with coronary artery calcification in patients without CKD or with normal renal function [12], [13].

Clinical studies have demonstrated conflicting evidence as to whether FGF23 imparts a protective or a harmful role on vasculature under stress. The relationship between FGF23 and the severity and extent of CAD based on coronary angiogram has not been characterized. Herein, we examined the relationship between circulating FGF23 concentration and the severity and extent of CAD in individuals who had undergone coronary angiography.

## Methods

### Ethics Statement

The study was approved by the Ethical Committee of Guangzhou General Hospital of Guangzhou Military Direct and the Ethical Committee of Zhujiang Hospital and the Ethical Committee of Sun Yat-Sen Memorial Hospital of Sun Yat-sen University. Written informed consent was obtained from all patients.

### Study Population

A total of 2,076 consecutive adult patients of both sexes (1263 male and 813 female, mean aged 62.5 years) who had undergone a diagnostic coronary angiography were enrolled from the Cardiology Department of Guangzhou General Hospital of Guangzhou Military Direct, Sun Yat-Sen Memorial Hospital and Zhujiang Hospital in Guangzhou, China, between December 2008 and September 2010. Inclusion criteria were: stable clinical condition except for acute coronary syndromes, and the availability of a coronary angiogram. The indications for angiography in individuals in clinically stable condition were chest pain and/or noninvasive test results consistent with myocardial ischemia. The exclusion criteria were critical illness or hemodynamic instability other than acute coronary syndromes, surgery or trauma within the previous month, known cancer, hepatic failure or hepatitis.

### Coronary Angiography

Coronary angiography was performed in all patients with a standard Judkins technique through the femoral artery or brachial artery. The angiograms were interpreted by two or more independent cardiologists who were unaware of the patients’ risk-factor profiles. All evaluations were performed based on the American Heart Association protocol [14]. The severity of CAD was evaluated either by determining the extent of coronary artery stenosis or measuring the number of stenotic vessels [15]. All patients were classified into four groups by coronary angiogram as follows: no stenosis, mild (<50%), moderate (50%–75%), and severe (>75%) stenosis in the major coronary arteries or their branches. Based on the number of stenotic main vessels, all patients were also divided into four additional groups: no stenosis, single-vessel disease (1-VD), two-vessel disease (2-VD) and three-vessel disease (3-VD). Furthermore, the coronary artery system was first divided into eight segments and a stenosis score was calculated by interventional cardiologists who were blinded to the study protocol as follows: left main stem, left anterior descending artery, diagonal branch, first septal perforator, left circumflex artery, main marginal branches, right coronary artery, and right posterior descending branch. The most severe stenosis in each of these segments was then scored, with 0 points for no stenosis, 1 point for 1% to 49% reduction in vessel diameter, 2 points for 50% to 74% reduction, 3 points for 75% to 99% stenosis, and 4 points for total occlusion of the segment. Scores for each segment were added and therefore the total scores could range from 0 to 32 points [16], [17]. This conventional scoring system was demonstrated to be significantly correlated with several other angiographic scoring systems and with atherosclerotic plaque burden [18].

### Intravascular Ultrasound (IVUS) Procedure and Examination

Coronary arterial plaque volumes and characteristics were analyzed by Gray-scale and virtual histology IVUS. Among 2076 patients undergoing coronary angiography, 372 patients received IVUS examination prior to percutaneous coronary artery intervention (PCI) for left anterior descending coronary artery, left circumflex coronary artery, or right coronary artery lesions with significant stenosis (defined as percent stenosis ≥50% diameter) as determined by quantitative coronary angiogram. For the IVUS procedure, a 30-MHz, 3.2-F, phased-array IVUS catheter (Volcano Corporation) was used. After placing the IVUS catheter at a point that was distant at least 30 mm from the coronary ostium, the catheter was pulled back to the coronary ostium with a motorized pull-back system at 1 mm/s. During pullback, gray-scale IVUS was recorded and raw radiofrequency data was captured at the top of the R-wave for reconstruction of the color-coded map by a virtual histology IVUS data recorded. IVUS core laboratory analyses were performed by an independent and experienced investigator in a blinded manner. Manual contour detection of both the lumen and the external elastic membrane (EEM) was performed for each frame. Quantitative IVUS gray-scale analysis was performed according to the guidelines of the American College of Cardiology and European Society of Cardiology [19]. The EEM volume and lumen volume were calculated, and the difference between the two values was defined as plaque volume. Virtual histology IVUS data analysis was carried out on the basis of a gray-scale border contour calculation, and the absolute value of each plaque component was measured automatically by the IVUSLab software (Volcano Corporation).

### End Points and Definitions

1045 of the 2076 patients (50.3%) had received coronary revascularization therapy including 914 patients undergoing PCI and 131 patients undergoing coronary artery bypass grafting (CABG). Clinical follow-up was performed at one year after coronary revascularization therapy by trained quality assurance nurses, who worked exclusively to determine clinical events via telephone contact or office visit. Clinical follow-up was available on all patients with revascularization therapy. The primary end points of the present study were the 12-month incidence of target vessel revascularization (TVR) and target lesion revascularization (TLR), as adjudicated by the independent clinical events committee. TLR was defined as either repeat percutaneous or surgical revascularization for a lesion anywhere within the stent or the 5-mm borders proximal or distal to the stent. TVR was considered to be driven by ischemia if the stenosis of the target vessel was at least 50% of the luminal diameter on the basis of a quantitative analysis, with either electrocardiographic changes while the patients was at rest or a functional study indicating ischemia in the distribution of the target vessel, or if there was stenosis of at least 70% in conjunction with recurrent symptoms alone [20].

### Baseline Investigation

Information on age, current smoking status, history of hypertension and diabetes, and use of medications was collected by questionnaire. The questionnaires were checked by a trained interviewer during a clinical visit. At the same visit, body weight, height, and blood pressure were measured by standard techniques in triplicate. Body mass index was calculated by dividing weight in kilograms by the height in square meters. Hypertension was defined as a systolic blood pressure >140 mm Hg and/or diastolic blood pressure >90 mm Hg, or patients who were receiving antihypertensive medication. Diabetes was considered to be present if there was a history of diabetes, a fasting blood glucose level >126 mg/dL, or if the patient was taking anti-diabetic medication.

### Biochemical Measurements

After the patients had fasted overnight, samples of venous blood were drawn into tubes before coronary angiography. Plasma and serum were separated from blood cells by immediate centrifugation and were divided into aliquots and stored at −80°C until analysis. Serum lipid levels (total cholesterol, triglycerides, and high-density lipoprotein (HDL) cholesterol), calcium, phosphate, and creatinine were measured by standard laboratory methods at our hospitals; low-density lipoprotein (LDL) cholesterol was calculated by the Friedewald formula [21]. We calculated estimated glomerular filtration rate (eGFR) with the Chronic Kidney Disease Epidemiology Collaboration equation (eGFR = 175 × standardized S_creatinine_^−1.154^ × age^−0.203^ × 1.212 [if black] × 0.742 [if female]), in which GFR is expressed as mL/min per 1.73 m^2^ of body surface area and S_creatinine_ is expressed in mg/dL [22]. Serum active intact full-length FGF23 was measured using a commercial sandwich ELISA according to the manufacturer’s protocol (Kainos Laboratories, Inc., Tokyo, Japan). This ELISA utilizes two murine MABs for two separate sites [23]. In this study, the intra- and inter-assay coefficients of variation were 3.7% and 4.5% respectively.

### Statistical Analyses

Data are presented as medians and interquartile ranges (IQR) for skewed variables. Unless otherwise indicated, values are expressed as mean ± SD or as percentages for categorical variables. Comparisons between groups were performed using Kruskal-Wallis test followed where relevant by Mann-Whitney *U* test or one-way analysis of variance with adjustment for multiple comparisons using Bonferroni correction method (continuous variables) or the chi-square test (categorical variables). Correlations between selected pairs of variables were evaluated with the spearman correlation. We performed a Kruskal-Wallis test to compute levels of FGF23 for different severity and extent of CAD. For this endeavor, values of circulating FGF23 were categorized into quartiles (cutoff values for FGF23 were 40.4, 44.5, and 51.3 pg/mL). Chi-square trend test was used to explore the frequency distribution of severity and extent of CAD across FGF23 quartiles. Furthermore, multivariate linear regression analysis was performed with the stenosis scores as dependent variable and log-transformed FGF23 as independent variable with adjusting for other covariates. Multiple logistic regression models were used to analyze the relationship between FGF23 and the incidence of TLR and TVR. Odds ratio (OR) and 95% confidence interval (CI) were presented. Two-side *P* values below 0.05 were considered to indicate statistical significance. All statistical analyses were performed using SPSS 13.0 software (SPSS Inc., Chicago, Illinois).

## Results

Serum FGF23 concentrations were skewed in distribution. The median serum FGF23 level in the whole sample was 44.5 pg/mL and IQR was from 40.4 to 51.3 pg/mL. Demographic and clinical characteristics of the study population across the quartiles of FGF23 concentrations are presented in Table 1. Serum FGF23 concentrations in male were higher than those in female (median 45.0 vs. 43.7 pg/mL, *P*<0.001). Higher serum FGF23 concentrations were associated with older age, higher body mass index, and diabetes in both male and female. There were no significant differences across the quartiles of serum FGF23 concentration by current smoking and hypertension. The proportions of participants who had used cholesterol-lowering agents, anti-diabetes drugs, and antihypertensive drugs were highest in the highest quartile and lowest in the lowest quartile of serum FGF23 (all *P*<0.001).

**Table 1:** Basic characteristics of the study population.

		FGF23 quartiles (pg/mL)				
	Whole sample	Q1 (<40.4)	Q2 (40.4–44.5)	Q3 (44.6–51.3)	Q4 (>51.3)	
Characteristic	n = 2076	n = 518	n = 520	n = 519	n = 519	*P*
Age, yrs	62.5±12.3	60.4±12.9	61.8±12.6	63.3±11.9	64.5±11.4	<0.001
Male sex	1263 (60.8)	301 (58.1)	301 (57.9)	314 (60.5)	347 (66.9)	0.003
BMI, kg/m^2^	24.4±4.76	23.9±5.04	24.1±3.91	24.0±4.57	25.7±5.20	<0.001
Diabetes	477 (23.0)	91 (17.6)	96 (18.5)	134 (25.8)	156 (30.1)	0.018
Hypertension	1268 (61.1)	305 (58.9)	316 (60.8)	314 (60.5)	333 (64.2)	0.104
Current smoker	460 (22.2)	118 (22.8)	110 (21.2)	101 (19.5)	131 (25.2)	0.485
**Medication use**						
Use of cholesterol-lowering agents	1238 (59.6)	254 (49.0)	288 (55.4)	349 (67.2)	347 (66.9)	<0.001
Use of anti-diabetic drugs	418 (20.1)	63 (12.2)	83 (16.0)	131 (25.2)	141 (27.2)	<0.001
Use of antihypertensive drugs	1248 (60.1)	256 (49.4)	288 (55.4)	350 (67.4)	354 (68.2)	<0.001
**Laboratory results**						
Triglycerides, mmol/L	1.55 (1.08–2.14)	1.56 (1.07–2.09)	1.50 (1.02–2.09)	1.54 (1.09–2.14)	1.64 (1.15–2.22)	0.227
Total cholesterol, mmol/L	4.74±1.06	4.70±1.08	4.73±1.03	4.74±1.02	4.78±1.11	0.628
LDL cholesterol, mmol/L	2.99±0.94	2.94±0.90	2.94±0.91	3.03±0.93	3.05±1.00	0.129
HDL cholesterol, mmol/L	1.08±0.32	1.09±0.30	1.13±0.31	1.07±0.29	1.01±0.36	<0.001
FGF23, pg/mL	44.5 (40.4–51.3)	37.9 (36.0–39.1)	42.4 (41.5–43.4)	46.9 (45.6–48.6)	73.8 (62.4–83.5)	–
Phosphate, mmol/L	1.14±0.24	1.12±0.22	1.13±0.23	1.11±0.21	1.21±0.30	<0.001
Calcium, mmol/L	2.38±0.18	2.39±0.16	2.38±0.17	2.36±0.18	2.40±0.21	0.018
eGFR, mL/min/1.73 m^2^	75.9 (65.1–91.1)	98.1 (93.4–107.8)	78.5 (73.0–85.4)	68.8 (63.4–75.3)	61.3 (53.9–73.9)	<0.001

Values are means ± SD, n (%), or median (interquartile range). BMI = body mass index, eGFR = estimated glomerular filtration rate, FGF23 = Fibroblast growth factor 23, HDL = high density lipoprotein, LDL = low density lipoprotein.

In univariate correlation analyses, serum FGF23 concentration was negatively correlated with HDL cholesterol (*r* = −0.146 and *r* = −0.145, *P*<0.001) and eGFR (*r* = −0.421 and *r* = −0.432, *P*<0.001) in both male and female. Furthermore, serum FGF23 concentration was positively correlated with serum calcium (*r* = 0.161 and *r* = 0.123, *P*<0.001) and phosphate (*r* = 0.252 and *r* = 0.258, *P*<0.001) in both male and female. However, weak correlation between serum FGF23 concentration and LDL cholesterol was only found in male. And there was no significant association of serum FGF23 concentration with total cholesterol and triglycerides (Table 2).

**Table 2:** Correlation coefficients between FGF23 and other characteristics.

	Male		Female	
	*r*	*P*	*r*	*P*
Age, yrs	0.115	**<0.001**	0.139	**<0.001**
BMI, kg/m^2^	0.199	**<0.001**	0.176	**<0.001**
Smokers	0.028	0.312	0.011	0.746
Hypertension	0.030	0.286	0.070	**0.047**
Diabetes	0.096	**0.001**	0.126	**<0.001**
Total cholesterol, mmol/L	0.040	0.158	0.026	0.464
Triglycerides, mmol/L	0.015	0.607	0.034	0.336
LDL cholesterol, mmol/L	0.058	**0.040**	0.026	0.452
HDL cholesterol, mmol/L	−0.146	**<0.001**	−0.145	**<0.001**
Phosphate, mmol/L	0.252	**<0.001**	0.258	**<0.001**
Calcium, mmol/L	0.161	**<0.001**	0.123	**<0.001**
eGFR, mL/min/1.73 m^2^	−0.421	**<0.001**	−0.432	**<0.001**
Stenosis scores	0.315	**<0.001**	0.291	**<0.001**

**Bold** *P* values are statistically significant. Abbreviations as in Table 1.

**Figure 1:**
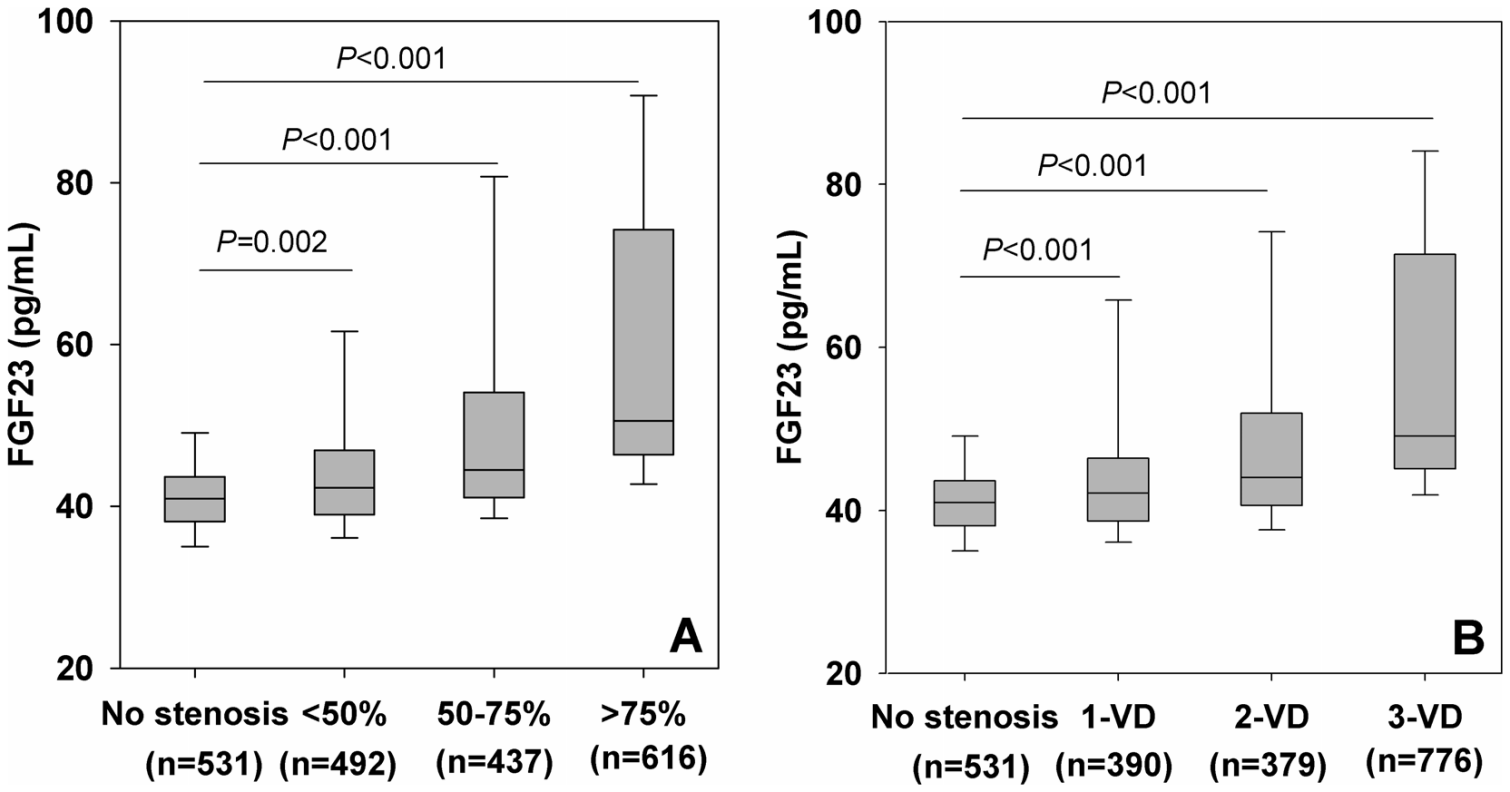
Box plots showing circulating FGF23 concentrations by angiographic extent of coronary artery disease. A. Severity of coronary artery stenosis. B. Number of stenosis vessels. Horizontal lines show median, 25th to 75th percentiles (boxes), and 10th to 90th percentiles (whiskers). FGF23 = Fibroblast growth factor 23, VD = vessel disease.

**Figure 2:**
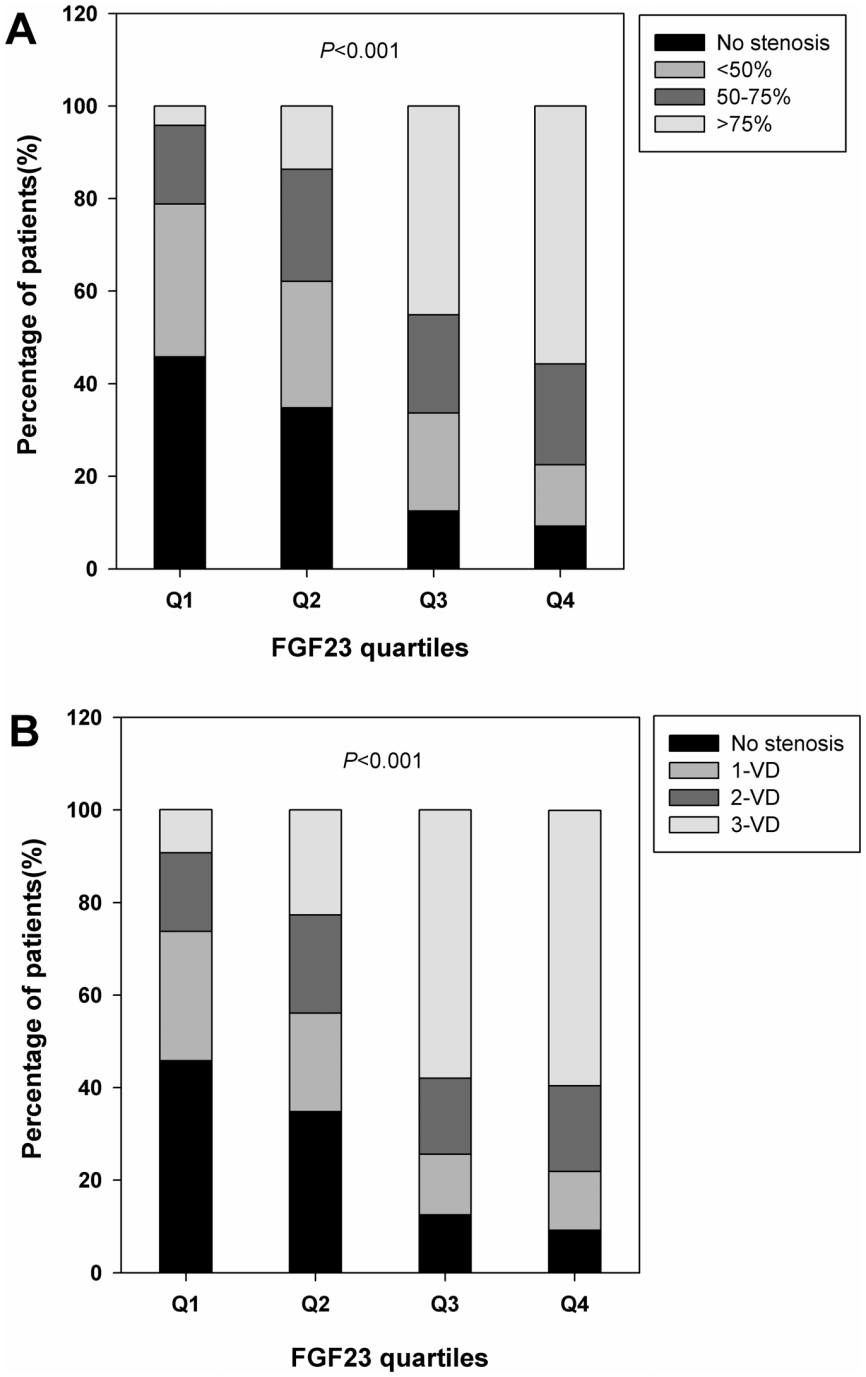
Frequency distribution of patients across FGF23 quartiles. A. Severity of coronary artery stenosis. B. Number of stenosis vessels. Abbreviations as in Figure 1.

To explore the relationship between serum FGF23 concentration and the severity and extent of CAD, we found a stepwise increase of serum FGF23 concentrations in patients with mild (median, 42.3 pg/mL; IQR, 38.9 to 46.9 pg/mL), moderate (median, 44.5 pg/mL; IQR, 41.1 to 53.7 pg/mL), severe stenosis (median, 50.6 pg/mL, IQR, 46.4 to 74.1 pg/mL) compared with those without stenosis (median, 40.9 pg/mL, IQR, 38.1 to 43.6 pg/mL), as shown in Figure 1A. Similarly, serum FGF23 concentrations were progressively increased across the number of stenotic vessels (Figure 1B). Furthermore, the proportions of patients who had no stenosis were highest in the lowest quartile and lowest in the highest quartile of serum FGF23 values (45.8% and 9.2%). By contrast, the proportions of patients who had severe stenosis or 3-VD were lowest in the lowest quartile and highest in the highest quartile of serum FGF23 values (4.2% and 55.7% or 9.3% and 59.5%, *P*<0.001) (Figure 2).

**Figure 3:**
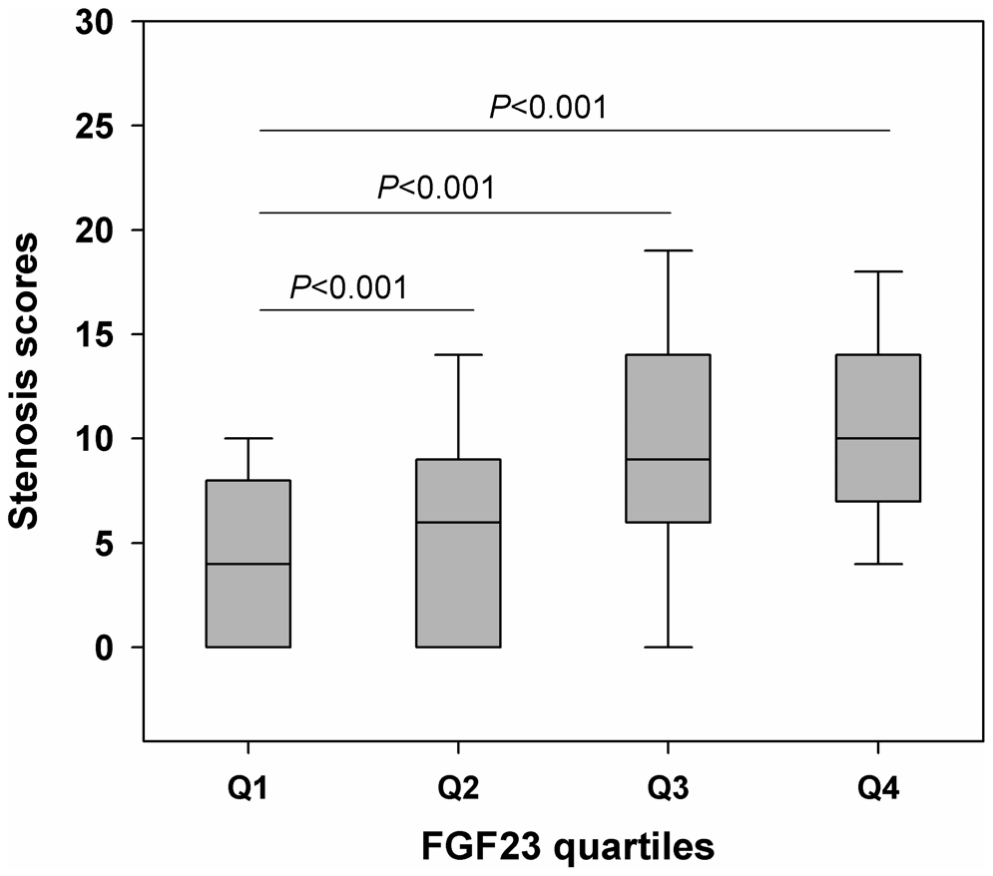
Box plots showing stenosis scores as the global index of severity and extent of coronary artery stenosis across FGF23 quartiles. Horizontal lines show median, 25th to 75th percentiles (boxes), and 10th to 90th percentiles (whiskers). Abbreviations as in Figure 1.

In addition, we combined the extent of stenosis and number of stenotic vessels into stenosis scores as the global index of the severity and extent of CAD, and found serum FGF23 concentration was positively correlated with stenosis scores in both male and female (*r* = 0.315 and *r* = 0.291, *P*<0.001) (Table 2). Furthermore, a total of 368 patients had an age-adjusted diminished renal function (eGFR<60 mL/min/1.73 m^2^), FGF23 was also significantly correlated with stenosis scores in both diminished and normal renal function (*r* = 0.355 and *r* = 0.288, *P*<0.001). Moreover, the stenosis scores were also gradually increased across the quartiles of serum FGF23 concentration (*P*<0.001) (Figure 3). To establish independent determinants of the stenosis scores, we performed linear regression analyses controlling for age, sex, body mass index, smokers, hypertension, diabetes, triglycerides, LDL cholesterol, HDL cholesterol, phosphate, calcium, eGFR, and serum FGF23 concentration in different models. In the final regression model that explained 16.3% (adjusted *R* ^2^ = 0.163, *P*<0.001) of the total variation of stenosis scores of the study population, serum FGF23 concentration was an independent strong predictor of the stenosis scores (standardized *β* = 0.326, *P*<0.001), as well as age, sex, body mass index, diabetes, and triglycerides (Table 3).

**Table 3:** Multivariate regression analysis of predictors affecting the stenosis scores.

	Model 1			Model 2			Model 3		
Independent Variables	β	t	*P*	β	t	*P*	β	t	*P*
FGF23, pg/mL*	0.342	16.459	**<0.001**	0.337	16.046	**<0.001**	0.326	13.322	**<0.001**
Age, yrs	0.157	7.643	**<0.001**	0.153	7.194	**<0.001**	0.154	7.238	**<0.001**
Sex	0.081	3.952	**<0.001**	0.072	3.193	**0.001**	0.070	3.077	**0.002**
BMI, kg/m^2^	0.041	1.975	**0.048**	0.042	2.074	**0.038**	0.042	2.068	**0.039**
Smokers				0.019	0.864	0.388	0.023	1.028	0.304
Hypertension				0.006	0.293	0.770	0.006	0.286	0.775
Diabetes				0.064	3.112	**0.002**	0.064	3.094	**0.002**
Triglycerides, mmol/L				0.074	2.984	**0.003**	0.073	2.950	**0.003**
LDL cholesterol, mmol/L				0.038	0.861	0.389	0.035	0.796	0.426
HDL cholesterol, mmol/L				0.026	1.062	0.288	0.024	0.974	0.330
Total cholesterol, mmol/L				−0.073	−1.528	0.127	−0.070	−1.473	0.141
Phosphate, mmol/L							−0.029	−1.397	0.163
Calcium, mmol/L							−0.013	−0.616	0.538
eGFR, mL/min/1.73 m^2^							−0.037	−1.609	0.108
R		0.396			0.408				0.404
R^2^		0.157			0.167				0.163

* Log-transformed variable; **Bold** *P* values are statistically significant. Abbreviations as in Table 1.

Next, we further analyzed the relationship between serum FGF23 level and coronary arterial plaque volumes and characteristics evaluated by IVUS. As shown in Table 4, plaque and dense calcium volumes were higher in patients with FGF23 in the highest quartile compared with the lowest quartile (mean ±SD, 426.7±188.5 vs. 353.1±163.1 mm^3^, and 21.4±16.2 vs.15.8±11.5 mm^3^, respectively). Correlation analyses showed serum FGF23 concentration was significantly correlated with plaque volume (*r* = 0.114, *P* = 0.028), average length (*r* = 0.112, *P* = 0.031), necrotic core volume (*r* = 0.121, *P* = 0.020), and dense calcium volumes (*r* = 0.107, *P* = 0.039).

**Table 4:** Gray-scale and virtual histology intravascular ultrasound results according to FGF23 quartiles.

		FGF23 quartiles (pg/mL)				
	All (n = 372)	Q1 (n = 111)	Q2 (n = 80)	Q3 (n = 94)	Q4 (n = 87)	*P*
**Gray-Scale IVUS**						
Average length, mm	46.1±22.9	41.6±20.5	48.6±23.7	46.2±25.6	49.5±21.4	0.067
External elastic membranevolume, mm^3^	869.9±412.4	832.3±375.2	817.3±448.4	902.2±480.7	931.3±331.3	0.523
Lumen volume, mm^3^	493.3±233.0	479.2±212.0	470.6±253.5	518.6±271.7	504.6±190.5	0.480
Plaque volume, mm^3^	376.7±190.1	353.1±163.1	346.7±194.9	383.6±209.0	426.7±188.5*	0.019
**Virtual Histology IVUS**						
Fibro-fatty volume, mm^3^	64.3±39.3	59.8±34.2	60.2±39.1	66.4±44.9	71.5±38.4	0.135
Fibrous tissue volume,mm^3^	159.7±98.6	152.5±87.2	157.3±98.6	171.2±121.4	158.7±84.5	0.590
Necrotic core volume,mm^3^	25.7±19.6	21.5±16.7	28.0±20.4	26.7±21.8	27.9±19.3	0.060
Dense calcium volume,mm^3^	18.2±14.6	15.8±11.5	17.4±12.7	18.6±17.3	21.4±16.2*	0.063

Values are means ± SD, *P<0.05 compared with quartile 1.

IVUS = intravascular ultrasound, other abbreviations as in Table 1.

**Figure 4:**
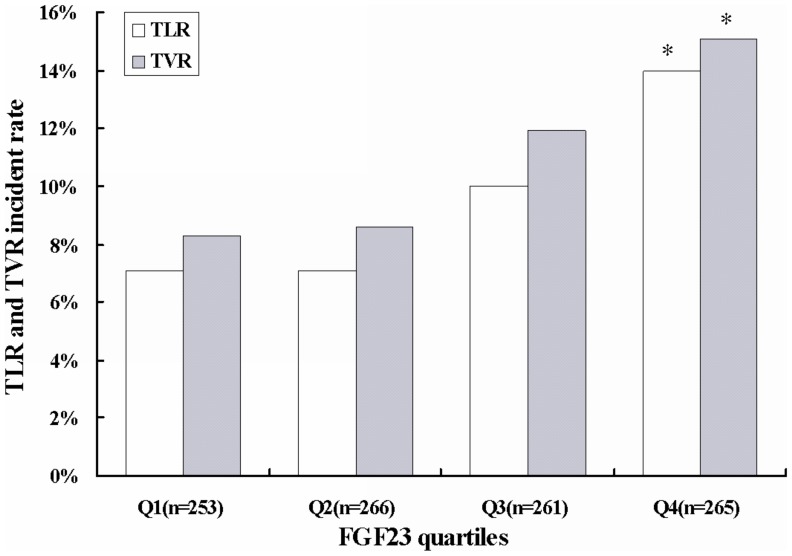
Incidence of TLR and TVR within one-year follow-up in patients undergoing coronary revascularization therapy according to FGF23 quartiles. *P<0.05 compared with quartile 1. TLR = target lesion revascularization, TVR = target vessel revascularization, other abbreviations as in Figure 1.

Analyses of 1-year follow-up data revealed that the patients with FGF23 in the highest quartile had significantly higher rates of TVR and TLR than those with FGF23 in the lowest quartile (15.1% vs.8.3%, and 14.0% vs.7.1%, respectively, both *P*<0.05) (Figure 4). Characteristics of target lesions and stents according to FGF23 quartiles were showed in Table S1. Only the degree of lesion calcification was found to be significantly different among the quartiles of FGF23. Multivariate analyses disclosed that the participants with FGF23 in the fourth quartile still had significantly higher rates of TVR and TLR than those in the first quartile after adjustment for all potential confounders (OR: 1.86, 95%CI: 1.02 to 3.41; and OR: 2.14, 95%CI: 1.13 to 4.05) (Table 5).

**Table 5:** Odds ratios for TLR and TVR according to quartiles of and log-transformed FGF23 level.

		FGF23 quartiles (pg/mL)				
	Per SD Increase	Q1 (n = 253)	Q2 (n = 266)	Q3 (n = 261)	Q4 (n = 265)	*P*
**TLR**						
Model 1	2.98 (1.60–5.56)†	1.00	1.01 (0.51–1.96)	1.44 (0.77–2.71)	2.12 (1.17–3.83)*	0.026
Model 2	2.99 (1.57–5.69)†	1.00	1.08 (0.55–2.13)	1.51 (0.79–2.85)	2.19 (1.18–4.09)*	0.038
Model 3	3.15 (1.55–6.42)*	1.00	1.07 (0.54–2.11)	1.48 (0.78–2.81)	2.14 (1.13–4.05)*	0.060
**TVR**						
Model 1	3.40 (1.89–6.11)†	1.00	1.04 (0.56–1.94)	1.48 (0.83–2.66)	1.96 (1.12–3.43)*	0.047
Model 2	3.52 (1.92–6.43)†	1.00	1.12 (0.59–2.09)	1.56 (0.86–2.81)	2.04 (1.14–3.64)*	0.057
Model 3	3.32 (1.70–6.47)*	1.00	1.12 (0.60–2.11)	1.53 (0.85–2.78)	1.86 (1.02–3.41)*	0.148

Values are OR (95%CI).

* *P*<0.05,

† *P*<0.001.

Model 1 adjusted for age, sex, and BMI. Model 2 adjusted for variables in model 1 plus smoking, hypertension, diabetes, triglycerides, total cholesterol, LDL cholesterol, and HDL cholesterol. Model 3 adjusted for variables in model 2 plus phosphate, calcium, eGFR, reference vessel diameter, lesion length, degree of lesion calcification, and stent types. SD = standard deviation, TLR = target lesion revascularization, TVR = target vessel revascularization, other abbreviations as in Table 1.

## Discussion

In the current study, we report an association between circulating FGF23 and the severity and extent of CAD in coronary angiographic patients. The association of FGF23 with severity and extent of CAD was existed significantly in both male and female and was particularly strong among the patients with eGFR<60 mL/min/1.73 m^2^. This significant association was independent of established risk factors of cardiovascular disease including age, body mass index, hypertension, diabetes, smoking, serum lipid levels, kidney function, and other abnormalities of mineral metabolism, such as serum phosphate level. Moreover, the association of severity of CAD with FGF23 was much stronger than those with the traditional cardiovascular risk factors. Furthermore, subgroup analyses showed FGF23 was significantly associated with plaque and dense calcium volumes evaluated by IVUS. Analyses of 1-year follow-up data showed that serum FGF23 levels were significantly independent predictors of TVR and TLR.

To our knowledge, only one previous study in a subsample of the Prospective Investigation of Vasculature in Uppsala Seniors (PIVUS study) has explored the relationship between FGF23 and total body atherosclerosis in the community population [10]. The results in our present study are consistent with the previous study. But there are some different places between our study and the PIVUS study. Firstly, the study subjects were 306 community elderly men and women, whereas the subjects of our study were the outpatients and inpatients with or without suspected CAD. Secondly, the PIVUS study used whole body magnetic resonance angiography to assess the total body atherosclerosis defined as the sum of vascular abnormalities for each of the five different vascular territories (neck, aorta, kidney, upper leg and lower leg). In contrast, we employed coronary angiography to estimate the severity and extent of coronary artery stenosis. Despite of the differences, both studies found a graded relationship between FGF23 and severity of atherosclerosis. Furthermore, endothelium dysfunction has been shown to precede atherosclerosis development and also predicts future cardiovascular risk [24]. Another study found that higher FGF23 levels were independently associated with impaired vasoreactivity and increased arterial stiffness in a recent community-based cohort with normal renal function and without derangements in mineral metabolism and was consistent with our current findings [9].

The potential role of FGF23 in the pathogenesis of atherosclerosis may be partly explained through its involvement in the complex process of vascular calcification. Vascular calcification is a common finding in coronary arteries and has long been known to occur as a part of the atherosclerotic process [25]. A large number of studies have shown that coronary luminal diameter stenosis assessed by coronary angiography was not associated with the subsequent occurrence of cardiovascular events in a linear relationship, but might be associated with coronary artery wall lesions, namely the stability of coronary atherosclerotic plaque [26]. The rupture of unstable plaques leads to coronary stenosis and acute coronary syndrome. Although calcified plaque has been considered as the established, stable, and quiescent atheroma [27], recent studies reported that spotty distribution of calcium in calcified plaque was an important characteristic of vulnerable plaque leading to plaque rupture, which was more frequently observed in the culprit lesions of patients with acute coronary syndrome [28], [29]. Higher levels of FGF23 were associated with the development of coronary artery calcification, particularly in the presence of chronic kidney disease [8], [30], [31]. In addition, a recent study found serum FGF23 levels were also associated with coronary calcification independent of classical cardiovascular risk factors in patients with suspected CAD and with preserved renal function [32]. Thus it is plausible to infer that FGF23 could be involved in the process of vascular calcification, since vascular calcification is associated with coronary artery stenosis, which may help explain the association between elevated FGF23 and the severity of coronary artery stenosis.

Circulating FGF23 concentrations are increased 2 to 5-fold above the normal range early in the course of kidney disease, but can reach 100-fold above normal levels in the end-stage renal disease [33]. Our results showed the association between FGF23 and the stenosis scores as the global index of the severity and extent of coronary artery stenosis was much stronger in the patients with eGFR<60 mL/min/1.73 m^2^ than those with normal renal function, which was consistent with the previous finding that the subgroup of individuals with eGFR<60 mL/min/1.73 m^2^ suffered from nearly a 6-fold increased odds of having a high atherosclerosis score when in the highest FGF23 tertile compared to the lowest [10]. However, a recent nested case-control study found that in the Health Professional Follow-Up cohort with normal kidney function and without cardiovascular disease at baseline, plasma FGF23 was not associated with the incident coronary heart disease [12]. It may be possible that FGF23 is associated with severity but not the incidence of cardiovascular disease or that increased FGF23 is primarily a risk factor for cardiovascular disease with impaired renal function.

In our patient population, FGF23 was significantly increased and associated with coronary stenosis independent of serum phosphorus level,which still remained within the normal range in majority of the population. Indeed, the increase in FGF23 concentration appears to be a compensatory mechanism to maintain serum phosphorus level in the normal range [33]. This adaptive compensatory mechanism inevitably linked to a reduction, not an increase in cardiovascular risk in hemodialysis patients [34]. Therefore, it is possible that elevated FGF23 level may be a response to, rather than a cause of, atherosclerosis. Further studies are needed to investigate the role of FGF23 in the process of atherosclerotic plaque formation.

There are some limitations in our study. First, as a cross-sectional study, the causal relationship between FGF23 concentration and the severity of coronary artery stenosis cannot be established. Second, we cannot rule out the possibility of residual confounding by other factors such as bone status, 1,25(OH)_2_D or PTH levels. Finally, prescribed or self-administered calcium and vitamin D supplements and dietary phosphate intake have not been considered in our calculations.

In conclusion, we report an independent association between circulating FGF23 concentration and the severity and extent of coronary artery stenosis in the coronary angiographic patients. Furthermore, FGF23 was also significantly associated with coronary arterial plaque volumes and characteristics and was an independent predictor of TLR and TVR at 1-year follow-up. Our observations provide an additional evidence of the role of FGF23 in cardiovascular disease. Future studies are needed to elucidate the potential biological mechanisms of FGF23 in the pathogenesis of cardiovascular disease and to evaluate whether FGF23 is a modifiable cardiovascular risk factor.

## Supporting Information

Table S1 **Baseline characteristics of lesions and stents according to FGF23 quartiles.**
(DOC)

## References

[1] Bhattacharyya N, Chong WH, Gafni RI, Collins MT (2012) Fibroblast growth factor 23: state of the field and future directions. Trends Endocrinol Metab 23: 610–618. 22921867 10.1016/j.tem.2012.07.002 PMC3502714

[2] Kurosu H, Ogawa Y, Miyoshi M, Yamamoto M, Nandi A, et al (2006) Regulation of fibroblast growth factor-23 signaling by klotho. J Biol Chem 281: 6120–6123. 16436388 10.1074/jbc.C500457200 PMC2637204

[3] Shimada T, Kakitani M, Yamazaki Y, Hasegawa H, Takeuchi Y, et al (2004) Targeted ablation of Fgf23 demonstrates an essential physiological role of FGF23 in phosphate and vitamin D metabolism. J Clin Invest 113: 561–568. 14966565 10.1172/JCI19081 PMC338262

[4] Palmer SC, Hayen A, Macaskill P, Pellegrini F, Craig JC, et al (2011) Serum levels of phosphorus, parathyroid hormone, and calcium and risks of death and cardiovascular disease in individuals with chronic kidney disease: a systematic review and meta-analysis. JAMA 305: 1119–1127. 21406649 10.1001/jama.2011.308

[5] Wolf M, Molnar MZ, Amaral AP, Czira ME, Rudas A, et al (2011) Elevated fibroblast growth factor 23 is a risk factor for kidney transplant loss and mortality. J Am Soc Nephrol 22: 956–966. 21436289 10.1681/ASN.2010080894 PMC3083317

[6] Kendrick J, Cheung AK, Kaufman JS, Greene T, Roberts WL, et al (2011) FGF-23 associates with death, cardiovascular events, and initiation of chronic dialysis. J Am Soc Nephrol 22: 1913–1922. 21903574 10.1681/ASN.2010121224 PMC3187186

[7] Parker BD, Schurgers LJ, Brandenburg VM, Christenson RH, Vermeer C, et al (2010) The associations of fibroblast growth factor 23 and uncarboxylated matrix Gla protein with mortality in coronary artery disease: the Heart and Soul Study. Ann Intern Med 152: 640–648. 20479029 10.1059/0003-4819-152-10-201005180-00004 PMC3079370

[8] Gutierrez OM, Januzzi JL, Isakova T, Laliberte K, Smith K, et al (2009) Fibroblast growth factor 23 and left ventricular hypertrophy in chronic kidney disease. Circulation 119: 2545–2552. 19414634 10.1161/CIRCULATIONAHA.108.844506 PMC2740903

[9] Mirza MA, Larsson A, Lind L, Larsson TE (2009) Circulating fibroblast growth factor-23 is associated with vascular dysfunction in the community. Atherosclerosis 205: 385–390. 19181315 10.1016/j.atherosclerosis.2009.01.001

[10] Mirza MA, Hansen T, Johansson L, Ahlstrom H, Larsson A, et al (2009) Relationship between circulating FGF23 and total body atherosclerosis in the community. Nephrol Dial Transplant 24: 3125–3131. 19429932 10.1093/ndt/gfp205

[11] Dalal M, Sun K, Cappola AR, Ferrucci L, Crasto C, et al (2011) Relationship of serum fibroblast growth factor 23 with cardiovascular disease in older community-dwelling women. Eur J Endocrinol 165: 797–803. 21873490 10.1530/EJE-11-0577 PMC3486640

[12] Taylor EN, Rimm EB, Stampfer MJ, Curhan GC (2011) Plasma fibroblast growth factor 23, parathyroid hormone, phosphorus, and risk of coronary heart disease. Am Heart J 161: 956–962. 21570529 10.1016/j.ahj.2011.02.012 PMC3095912

[13] Roos M, Lutz J, Salmhofer H, Luppa P, Knauss A, et al (2008) Relation between plasma fibroblast growth factor-23, serum fetuin-A levels and coronary artery calcification evaluated by multislice computed tomography in patients with normal kidney function. Clin Endocrinol (Oxf) 68: 660–665. 17970775 10.1111/j.1365-2265.2007.03074.x

[14] Austen WG, Edwards JE, Frye RL, Gensini GG, Gott VL, et al (1975) A reporting system on patients evaluated for coronary artery disease. Report of the Ad Hoc Committee for Grading of Coronary Artery Disease, Council on Cardiovascular Surgery, American Heart Association. Circulation 51: 5–40. 1116248 10.1161/01.cir.51.4.5

[15] Pajunen P, Nieminen MS, Taskinen MR, Syvanne M (1997) Quantitative comparison of angiographic characteristics of coronary artery disease in patients with noninsulin-dependent diabetes mellitus compared with matched nondiabetic control subjects. Am J Cardiol 80: 550–556. 9294980 10.1016/s0002-9149(97)00420-7

[16] Budde T, Fechtrup C, Bosenberg E, Vielhauer C, Enbergs A, et al (1994) Plasma Lp(a) levels correlate with number, severity, and length-extension of coronary lesions in male patients undergoing coronary arteriography for clinically suspected coronary atherosclerosis. Arterioscler Thromb Vasc Biol 14: 1730–1736. 10.1161/01.atv.14.11.1730 7947596

[17] Jenkins PJ, Harper RW, Nestel PJ (1978) Severity of coronary atherosclerosis related to lipoprotein concentration. Br Med J 2: 388–391. 210876 10.1136/bmj.2.6134.388 PMC1609066

[18] Neeland IJ, Patel RS, Eshtehardi P, Dhawan S, McDaniel MC, et al.. (2012) Coronary angiographic scoring systems: an evaluation of their equivalence and validity. Am Heart J 164: 547–552 e541. 10.1016/j.ahj.2012.07.007 PMC3913177 23067913

[19] Mintz GS, Nissen SE, Anderson WD, Bailey SR, Erbel R, et al (2001) American College of Cardiology Clinical Expert Consensus Document on Standards for Acquisition, Measurement and Reporting of Intravascular Ultrasound Studies (IVUS). A report of the American College of Cardiology Task Force on Clinical Expert Consensus Documents. J Am Coll Cardiol 37: 1478–1492. 11300468 10.1016/s0735-1097(01)01175-5

[20] Stone GW, Ellis SG, Cox DA, Hermiller J, O’Shaughnessy C, et al (2004) A polymer-based, paclitaxel-eluting stent in patients with coronary artery disease. N Engl J Med 350: 221–231. 14724301 10.1056/NEJMoa032441

[21] Friedewald WT, Levy RI, Fredrickson DS (1972) Estimation of the concentration of low-density lipoprotein cholesterol in plasma, without use of the preparative ultracentrifuge. Clin Chem 18: 499–502. 4337382

[22] Levey AS, Stevens LA, Schmid CH, Zhang YL, Castro AF III, et al (2009) A new equation to estimate glomerular filtration rate. Ann Intern Med 150: 604–612. 19414839 10.7326/0003-4819-150-9-200905050-00006 PMC2763564

[23] Yamazaki Y, Okazaki R, Shibata M, Hasegawa Y, Satoh K, et al (2002) Increased circulatory level of biologically active full-length FGF-23 in patients with hypophosphatemic rickets/osteomalacia. J Clin Endocrinol Metab 87: 4957–4960. 12414858 10.1210/jc.2002-021105

[24] Davignon J, Ganz P (2004) Role of endothelial dysfunction in atherosclerosis. Circulation 109: III27–32. 15198963 10.1161/01.CIR.0000131515.03336.f8

[25] Frink RJ, Achor RW, Brown AL Jr, Kincaid OW, Brandenburg RO (1970) Significance of calcification of the coronary arteries. Am J Cardiol 26: 241–247. 5505449 10.1016/0002-9149(70)90790-3

[26] Sangiorgi G, Rumberger JA, Severson A, Edwards WD, Gregoire J, et al (1998) Arterial calcification and not lumen stenosis is highly correlated with atherosclerotic plaque burden in humans: a histologic study of 723 coronary artery segments using nondecalcifying methodology. J Am Coll Cardiol 31: 126–133. 9426030 10.1016/s0735-1097(97)00443-9

[27] Abedin M, Tintut Y, Demer LL (2004) Vascular calcification: mechanisms and clinical ramifications. Arterioscler Thromb Vasc Biol 24: 1161–1170. 15155384 10.1161/01.ATV.0000133194.94939.42

[28] Ehara S, Kobayashi Y, Yoshiyama M, Shimada K, Shimada Y, et al (2004) Spotty calcification typifies the culprit plaque in patients with acute myocardial infarction: an intravascular ultrasound study. Circulation 110: 3424–3429. 15557374 10.1161/01.CIR.0000148131.41425.E9

[29] Kataoka Y, Wolski K, Uno K, Puri R, Tuzcu EM, et al (2012) Spotty calcification as a marker of accelerated progression of coronary atherosclerosis: insights from serial intravascular ultrasound. J Am Coll Cardiol 59: 1592–1597. 22538329 10.1016/j.jacc.2012.03.012

[30] Lim K, Lu TS, Molostvov G, Lee C, Lam FT, et al (2012) Vascular Klotho deficiency potentiates the development of human artery calcification and mediates resistance to fibroblast growth factor 23. Circulation 125: 2243–2255. 22492635 10.1161/CIRCULATIONAHA.111.053405

[31] Desjardins L, Liabeuf S, Renard C, Lenglet A, Lemke HD, et al (2012) FGF23 is independently associated with vascular calcification but not bone mineral density in patients at various CKD stages. Osteoporos Int 23: 2017–2025. 22109743 10.1007/s00198-011-1838-0

[32] Masai H, Joki N, Sugi K, Moroi M (2012) A preliminary study of the potential role of FGF-23 in coronary calcification in patients with suspected coronary artery disease. Atherosclerosis: doi:10.1016/j.atherosclerosis.2012.1010.1045. 10.1016/j.atherosclerosis.2012.10.045 23137826

[33] Fliser D, Kollerits B, Neyer U, Ankerst DP, Lhotta K, et al (2007) Fibroblast growth factor 23 (FGF23) predicts progression of chronic kidney disease: the Mild to Moderate Kidney Disease (MMKD) Study. J Am Soc Nephrol 18: 2600–2608. 17656479 10.1681/ASN.2006080936

[34] Block GA, Klassen PS, Lazarus JM, Ofsthun N, Lowrie EG, et al (2004) Mineral metabolism, mortality, and morbidity in maintenance hemodialysis. J Am Soc Nephrol 15: 2208–2218. 15284307 10.1097/01.ASN.0000133041.27682.A2

