# Supplementary material for: Circulating Fibroblast Growth Factor 23 Is Associated with Angiographic Severity and Extent of Coronary Artery Disease
Source: PLoS One. 2013 Aug 28;8(8):e72545. doi: 10.1371/journal.pone.0072545 (PMC3755980; doi:10.1371/journal.pone.0072545)
Supplement: Table S1 — Baseline characteristics of lesions and stents according to FGF23 quartiles. (DOC) [file pone.0072545.s001.doc]

**Table S1** **Baseline characteristics of lesions and stents according to FGF23 quartiles**

|  |  | **FGF23 quartiles (pg/mL)** | | | |  |
| --- | --- | --- | --- | --- | --- | --- |
|  | **All (n=1045)** | **Q1 (n=253)** | **Q2 (n=266)** | **Q3 (n=261)** | **Q4 (n=265)** | ***P*** |
| Reference vessel diameter (mm) | 2.66±0.36 | 2.65±0.38 | 2.69±0.39 | 2.67±0.36 | 2.65±0.37 | 0.619 |
| Lesion length (mm) | 13.3±5.5 | 12.8±5.1 | 13.2±4.8 | 13.5±5.8 | 13.7±6.1 | 0.275 |
| Degree of lesion calcification (%) |  |  |  |  |  |  |
| No calcification | 56.4 | 60.1 | 55.3 | 62.5 | 47.9 | 0.044 |
| Mild calcification | 15.4 | 17.0 | 15.8 | 13.0 | 15.8 |  |
| Moderate calcification | 15.1 | 13.0 | 15.4 | 12.6 | 19.2 |  |
| Severe calcification | 13.1 | 9.9 | 13.5 | 11.9 | 17.0 |  |
| Stent types (%) |  |  |  |  |  |  |
| BMS | 26.3 | 28.5 | 24.4 | 25.7 | 26.8 | 0.059 |
| DES | 61.1 | 64.4 | 63.5 | 57.9 | 58.9 |  |

Values are means ± SD and %. BMS = bare metal stent, DES = drug-eluting stent.
